# Regulation of the Na^+^/K^+^-ATPase Ena1 Expression by Calcineurin/Crz1 under High pH Stress: A Quantitative Study

**DOI:** 10.1371/journal.pone.0158424

**Published:** 2016-06-30

**Authors:** Silvia Petrezsélyová, María López-Malo, David Canadell, Alicia Roque, Albert Serra-Cardona, M. Carmen Marqués, Ester Vilaprinyó, Rui Alves, Lynne Yenush, Joaquín Ariño

**Affiliations:** 1 Institut de Biotecnologia i Biomedicina, Universitat Autònoma de Barcelona, Cerdanyola del Vallès 08193, Barcelona, Spain; 2 Departament de Bioquímica i Biologia Molecular, Universitat Autònoma de Barcelona, Cerdanyola del Vallès 08193, Barcelona, Spain; 3 Instituto de Biología Molecular y Celular de Plantas (IBMCP), Universitat Politècnica de València-Consejo Superior de Investigaciones Científicas, Valencia, 46022, Spain; 4 IRB Lleida, Universitat de Lleida, Lleida 25198, Spain; 5 Universitat de Lleida, Lleida 25198, Spain; Kinki University School of Pharmaceutical Sciences, JAPAN

## Abstract

Regulated expression of the Ena1 Na^+^-ATPase is a crucial event for adaptation to high salt and/or alkaline pH stress in the budding yeast *Saccharomyces cerevisiae*. *ENA1* expression is under the control of diverse signaling pathways, including that mediated by the calcium-regulatable protein phosphatase calcineurin and its downstream transcription factor Crz1. We present here a quantitative study of the expression of Ena1 in response to alkalinization of the environment and we analyze the contribution of Crz1 to this response. Experimental data and mathematical models substantiate the existence of two stress-responsive Crz1-binding sites in the *ENA1* promoter and estimate that the contribution of Crz1 to the early response of the *ENA1* promoter is about 60%. The models suggest the existence of a second input with similar kinetics, which would be likely mediated by high pH-induced activation of the Snf1 kinase.

## Introduction

Excessive intracellular concentration of sodium cations is deleterious for most eukaryotic cells and, therefore, diverse mechanisms to avoid cytosolic accumulation of this cation have been evolutionarily developed. For instance, the yeast *Saccharomyces cerevisiae*, often used as a model to study the mechanisms of cation homeostasis, resorts to both sequestration of sodium into organelles (mainly the vacuole) and to extrusion of this cation through transporters located at the cell membrane [1].

Extrusion of sodium is achieved by two main mechanisms. The first one is based on the H^+^/Na^+^ antiporter encoded by *NHA1*, which exchanges protons with Na^+^, Li^+^, and K^+^ cations, and has major biological significance under normal (acidic) growth conditions [2]. The second strategy is based on a P-type ATPase pump encoded by the ENA system, which becomes essential for growth particularly at pHs above neutrality. *ENA* genes are found exclusively in fungi, bryophyta and protozoa and *ENA* homologues are likely to exist in all fungal genomes [3]. In most budding yeast strains *ENA* genes exist as a cluster of 3 to 5 elements encoding nearly identical proteins. The relevance of Ena1 in the tolerance to increased extracellular levels of toxic monovalent cations (Na^+^ and Li^+^) or alkaline pH is documented by the extreme sensitivity of cells lacking functional *ENA* genes under these circumstances (see [4] and references therein). Whereas the function of a Na^+^-ATPase pump is evident in response to high salt, the role of such protein under high pH stress, even in the absence of elevated sodium levels in the medium, became evident only recently [5]. This report demonstrated that Ena1 is required to pump out the sodium cations introduced by the Na^+^/phosphate cotransporter Pho89, whose role in phosphate uptake becomes more relevant at high external pH [6,7].

Under standard growth conditions (acidic pH and absence of osmotic stress) expression of the *ENA* genes is minimal. However, addition of NaCl above 0.3–0.4 M or raising the external pH above 7.2 results in rapid induction of the first member of the cluster, usually denoted as *ENA1* [8–11]. Work during the last twenty years has revealed the complex regulatory network that represses *ENA1* under normal growth conditions and promotes rapid increase of *ENA1* mRNA levels upon salt stress or high pH. This network includes the Rim101, Snf1, PKA and calcineurin pathways (see [12] and references therein). In addition to these pathways, activation of the osmosensitive Hog1 pathway becomes an important component of the response to high salt [13]. However, diverse evidences demonstrate that the Hog1 pathway does not contribute to either high pH stress tolerance nor to *ENA1* induction in response to high pH stress [10,14].

Work in our laboratory [14] illustrated the relative contribution of the calcineurin, Snf1 and Rim101 pathways to the induction of the *ENA1* gene in response to high pH. Although the analysis was semi-quantitative, it became evident that the calcineurin pathway was an important contributor to the alkaline responsiveness of *ENA1*. In budding yeast calcineurin (also known as protein phosphatase type 2B) is a heterodimeric Ser/Thr phosphatase composed of one of two possible redundant catalytic subunits (Cna1 and Cna2) plus a unique regulatory subunit (Cnb1). Calcineurin is activated by calcium and calmodulin. In response to alkaline pH stress, activation of calcineurin occurs as a consequence of the almost immediate entry of extracellular calcium through the Cch1-Mid1 calcium channel [15], and it affects the expression of a substantial number of genes [16,17]. The transcriptional effects of calcineurin activation occur mainly through the dephosphorylation of the transcription factor Crz1/Tcn1/Hal8

[18–20]. Dephosphorylated Crz1 enters the nucleus via the importin Nmd5 [21] and binds to its target promoters via specific sequences, known as CDREs (Calcineurin Dependent Response Elements). After phosphorylation, the transcription factor returns to the cytosol via the exportin Msn5 [22]. It has been proposed that regulation of *ENA1* in response to calcineurin activation would involve two functional Crz1-binding regions in the *ENA1* promoter at positions -813/-821 and -719/-727. The more upstream element (relative to the initiating Met codon) binds Crz1 with lesser affinity and it has been proposed to be responsible for the basal *ENA1* expression [23].

Despite early reports suggesting posttranscriptional regulation of Ena1 activity [24] and the evidence of diverse components necessary for the correct localization of Ena1 at the plasma membrane [25,26], it is widely accepted that regulation of Ena1 function in response to stress occurs principally by regulation of the mRNA levels. It has been assumed that changes in *ENA1* mRNA levels occur mainly in response to modulation of *ENA1* promoter activity. However, recent work has showed that, after alkaline stress, the modification of mRNA levels for many genes is largely dominated by changes in mRNA stability [27]. In this work we address, in a quantitative study, the different steps relevant for the expression of Ena1 in response to high pH stress and, in particular, the contribution of the Crz1 transcription factor in this process.

## Materials and Methods

### Yeast strains and growth conditions

*S*. *cerevisiae* strains used in this study were all derived from BY4741 (*MAT*a *his3Δ*1 *leu2Δ*0 *met15Δ*0 *ura3Δ*0; obtained from EUROSCARF), which has an S288C background. Strain SP020, which expresses the Crz1 protein C-terminally tagged with the 3xHA epitope was previously described [5]. Strains carrying a Crz1 (SP017) or Ena1 (MLM001) protein C-terminally tagged with GFP epitope were constructed by transformation of the wild type strain with a cassette amplified from plasmid pFA6-GFP-*kanMX6* (for Crz1) or pFA6-GFP(S65T)-*HIS3MX6* (for Ena1) [28] using oligonucleotides CRZ1-C-pFA6-dir/CRZ1-C-pFA6-rev and ENA1-C-pFA6-dir/ENA1-C-pFA6-rev, respectively. The *ENA1* gene was tagged similarly in the *crz1*::*kanMX4* background to generate strain MLM002. Correct integration was verified by diagnostic PCR. Since members of the *ENA* cluster are highly similar, care was taken to select the transformants carrying specifically a C-terminally GFP tagged version of *ENA1*. To this end, two pairs of primers were used: ENA1-pFA6-comp-Cter and pFA6a_GFP_rev that amplify a region regardless the member of *ENA* cluster receiving the tag, and ENA1-pFA6-comp-Nter and pFA6a_GFP_rev that amplify a specific fragment for *ENA1* integration. Oligonucleotides used in this work are described in S1 Table.

Strain SP039 used for nuclear colocalization studies and quantification of Crz1 in single cells by confocal microscopy was generated by crossing SP017 (BY4741 *CRZ1*:GFP-*kanMX6*) strain to BY4742 *SIK1*:mRFP-*kanMX6* [29] and subsequent separation of tetrads by micromanipulation. The correct genotype of clones carrying both mutations was verified by diagnostic PCR analysis and analyzed for positive GFP and RFP signals by fluorescence microscopy. Strain BY4742 *SIK1*:mRFP-*kanMX6* was a gift of H. Sychrova (Institute of Physiology, Prague). Strains employed in this work are described in Table 1.

**Table 1:** Strains used in this study.

Strain	Genotype	Source /Reference
BY4741	*MAT*a *his3Δ*1 *leu2Δ*0 *met15Δ*0 *ura3Δ*0	[67]
SP017	BY4741 *CRZ1*-GFP-*kanMX6*	This work
SP020	BY4741 *CRZ1*-3XHA-*kanMX6*	[5]
MLM001	BY4741 *ENA1*-GFP-*HIS3*	This work
MLM002	BY4741 *crz1*:: *kanMX4 ENA1*-GFP-*HIS3*	This work
SP039	BY4741 *CRZ1*-GFP-*kanMX6 SIK1*-mRFP-*kanMX6*	This work
*SIK1*-mRFP	BY4742 *SIK1*-mRFP-*kanMX6*	[29]

To induce alkaline pH stress response, *S*. *cerevisiae* strains were usually grown overnight to mid-exponential phase at 28°C in liquid rich medium (YPD; 10 g/l yeast extract, 20 g/l peptone and 20 g/l dextrose) and then resuspended in fresh YPD media containing 50 mM TAPS adjusted to pH 5.5 to OD_600_ = 0.2. Cells were grown for 4–5 hours to reach OD_600_ = 0.6–0.8 and treated with 1 M KOH to reach external pH 8.0. Samples (10–40 ml) were taken at the appropriate times and then either harvested by centrifugation (directly or after formaldehyde treatment for ChIP or ChIP-Seq) or, for RNA extraction, by filtration on 0.45 m GN-6 filters (Pall Co.). When subcellular localization of Crz1-GFP was inspected by either confocal microscopy or fluorescence microscopy in combination with a microfluidic device, SP039 and SP017 cells respectively, were cultivated in a low-fluorescent minimal medium lacking riboflavin and folic acid [30] containing 50 mM TAPS, and high pH stress was initiated as described above. For immunoblot analysis of Ena1-GFP, cells were grown overnight in YPD at 28°C in liquid medium and then resuspended in 250 ml of fresh YPD containing 50 mM TAPS adjusted to pH 5.5 to OD_600_ = 0.1. Cells were grown to reach OD_600_ = 0.4 and then treated with 5 M KOH.

### Plasmids and recombinant DNA techniques

*Escherichia coli* DH5α strain was used as plasmid DNA host and grown in Luria-Bertani (LB) broth at 37°C. If necessary, *E*. *coli* cultures were supplemented with ampicillin and/or chloramphenicol to a final concentration of 100 μg/ml and 34 μg/ml, respectively. Recombinant techniques and bacterial and yeast transformations were carried out by standard methods. Plasmid pLMB127 [22], containing the triple GFP fusion to N-terminus of Crz1, was a generous gift of M. Cyert (University of Stanford). Plasmid pGEX-6P1-3HA-YPI1 was constructed as follows. The DNA fragment containing *YPI1* ORF, N-terminally tagged with 3 copies of the HA (hemagglutinin) epitope, was removed from pWS-Ypi1 [31] by BglII and SalI digestion and subcloned into the BamHI/SalI site of pGEX-6P-1 to express a GST-3HA-Ypi1 fusion protein in *E*. *coli*. The proper fusion of GST to 3HA-*YPI1* DNA sequence was confirmed by restriction and DNA sequencing analysis.

### Expression and purification of recombinant GST-3HA-Ypi1 protein in *E*. *coli*

Purification of the fusion protein GST-3HA-Ypi1 was carried out as described in [31], with some modifications. *E*. *coli* BL21-Codon Plus (DE3)-RIL cells were transformed with pGEX-6P1-3HA-YPI1 and grown overnight at 37°C in LB medium containing ampicillin and chloramphenicol. The culture was then diluted in the same medium to grow about 4 hours at 37°C to reach OD_600_ 0.3, isopropyl-1-β-D-thiogalactopyranoside was added to a final concentration of 0.1 mM, and culture was grown overnight at 26°C. Cells were harvested and resuspended in ice-cold lysis buffer (50 mM Tris-HCl pH 7.5, 150 mM NaCl, 10% glycerol, 0.1% Triton X-100, 2 mM DTT, 0.5 mM PMSF (phenylmethylsulfonyl fluoride) and complete protease inhibitor mixture (Roche Applied Science)). Cells were ruptured using a Bioruptor Plus UCD-300 equipment (Diagenode) provided with a cooling system (4°C) for 10 cycles (high intensity; 30 s of sonication followed by 30 s pause). The fusion protein was purified by incubating the bacterial crude lysate with Glutathione Sepharose resin (ABT beads, #4B-GLU-10, 50% solution in PBS) overnight at 4°C with gentle shaking. GST-3HA-Ypi1 fusion protein was eluted with 10 mM reduced glutathione and aliquots were stored at -80°C. The amounts of the eluted fusion protein were quantified using protein gels. Three different amounts of each protein eluate were loaded on an SDS/PAGE gel alongside with serial dilutions of BSA (Sigma-Aldrich) ranging from 0.6 to 2 μg. The Coomassie Brilliant Blue stained gel was scanned and the Gel Analyzer software (http://gelanalyzer.com/) used to quantify the amount of GST-3HA-Ypi1 by comparison of the staining intensity of the bands corresponding to the fusion protein and the BSA bands.

### Preparation of total protein yeast extracts

For Crz1-3HA immunodetection, whole cell protein extracts were prepared by harvesting 10 ml of yeast cultures (OD_600_ ~ 0.7 at t = 0), whereas for Ena1-GFP the appropriate volume was centrifuged to harvest ~12 OD_600_ units of cells. In both cases cells were resuspended in ice-cold lysis buffer (50 mM Tris-HCl pH 7.5, 150 mM NaCl, 0.1% Triton X-100, 10% glycerol, 1 mM DTT, 2 mM PMSF and complete protease inhibitor mixture (Roche Applied Science)). Zirconia/Silica beads (0.1 g, 0.5 mm diameter; #11079105z, BioSpec) were added to the suspensions, and then cells were ruptured using FastPrep^®^-24 (MP Biomedicals) with potency 5.5 for ~ 2 min. The lysates were centrifuged at 500 x g for 10 min at 4°C and aliquots of the supernatants stored at -80°C.

### Quantitative immunoblotting

The amounts of Crz1-3xHA and Ena1-GFP were determined by quantitative immunoblotting by comparing the Crz1 signal with GST-3HA-Ypi1 prepared and quantified as described above and the Ena1-GFP signal with recombinant GFP (rGFP) standard (#MB0752; Vector Laboratories, Inc.). Calibration standards were prepared by diluting rGFP or GST-3HA-Ypi1 into total protein extracts (described above) from the relevant strains to provide a range of the standard from 0.22 to 1.33 ng (Ypi1-3HA) and 2.5 to 10 ng (rGFP). 80 μg (for Ena1-GFP) or 70 μg (for Crz1-HA) of total protein were loaded on 10% (Ena1) or 8% (Crz1) SDS gels, electrophoresed and transferred to Immobilon^®^-P PVDF Transfer Membrane (Millipore). To ensure the homogeneity of the process, each complete experiment was contained in a single gel. Membranes were incubated overnight with anti-GFP (#11 814 460 001; Roche Applied Science) or with anti-HA (#11 583 816 001; clone 12CA5; Roche Applied Science) at 1:1000 dilution to detect Ena1-GFP and Crz1-3xHA, respectively. A 1:10000 dilution of horseradish peroxidase-conjugated anti-mouse antibody was used to detect primary antibodies. HA immunocomplexes were visualized by the ECL Prime Western Blotting Detection kit (GE Healthcare Life Sciences) and GFP immunocomplexes were visualized by the WESTAR Supernova kit (Cyagen). The chemiluminescence was recorded with a VersaDoc^TM^ 4000 MP imaging system (BioRad). GelAnalyzer software was used to quantify signal intensities.

### Chromatin Immunoprecipitation (ChIP) and ChIP-Sequencing (ChIP-Seq) assays

ChIP experiments were performed as described previously [5]. The biological material for ChIP-Seq was prepared essentially as for ChIP with the following exceptions: i) chromatin was fragmented using a Bioruptor Plus UCD-300 equipment (Diagenode) provided with a cooling system (4°C) for 30 cycles (high intensity; 30 s of sonication followed by 60 s pause) to generate fragments of ≤ 500 bp length; ii) the supernatant was pre-cleared with protein G sepharose^TM^ fast flow (GE Healthcare, #17-0618-01) beads for 1 h at 4°C and then, the pre-cleared cell lysate was incubated with 1 μg of polyclonal anti-HA ChIP-grade (Abcam, #ab9110) antibody overnight at 4°C; iii) anti-HA-Crz1-DNA complexes were collected with protein-G-sepharose beads incubating for 1 hour at 4°C and iv) after reverse crosslinking, DNA was extracted using a NucleoSpin^®^ Gel and PCR Clean-up kit (Macherey Nagel, #740609) and NTB buffer (Macherey-Nagel, #740595); v) recovered DNA (40 ng/reaction) was subjected to PCR using oligonucleotides that recognize various regions of *ENA1* promoter sequences. Oligonucleotide primer sequences are given in S1 Table. ChIP libraries were prepared using the TruSeq ChIP Sample Preparation Kit (Illumina) and then subjected to paired-end deep sequencing with the MiSeq Reagent Kit v2 (300 cycle) to provide reads of around 150 nt. FASTQ files were mapped using Bowtie2 (version 2.1.0) to generate the corresponding SAM files. Mapped reads (1.9 to 3.7 million/time point) were sorted and indexed using IGV tools [32]. Subsequent analysis was performed using the SeqMonk software (Babraham Institute, http://www.bioinformatics.babraham.ac.uk/projects/seqmonk/). The *S*. *cerevisiae* EF4 genomic data was employed. The chromosomal coordinates of the intergenic regions and information on the flanking genes were obtained from the Saccharomyces Genome Database using the YeastMine tool. This information was loaded into SeqMonk as an Annotation set and was used to generate 6303 intergenic probes. Subsequently, each of these probes were tiled in contiguous sections of 50 nt. This generated a total number of 65198 probes that were quantified by the read count quantification method, with identical reads removed and correction for total read count referred to the largest Dataset.

### RNA preparation and cDNA synthesis

Total RNA was purified using the RiboPure Yeast kit (Ambion; #AM1926) following the manufacturer’s instructions. RNA quality was assessed by electrophoresis in a denaturing 1.2% agarose FlashGel RNA cassette (Lonza). RNA concentrations were determined using a NanoDrop ND-1000 spectrophotometer (NanoDrop Technologies). The RNA was reverse-transcribed with the iScript cDNA synthesis kit (Bio-Rad; #170–8890) following the manufacturer’s guidelines. The reaction contains 500 ng of total RNA as a template in a reaction volume of 10 μl.

### qPCR assay

Real time quantitative PCR (qPCR) was performed using the SsoAdvanced^TM^ SYBR^®^ Green Supermix kit (BioRad; #172–5260) in 25 μl QPCR reaction according to the manufacturer’s protocol in a 96-well plate (4titude Ltd. United Kingdom; #4ti-0720/C). The samples were amplified using a CF96^TM^ Real-Time PCR System (BioRad). For quantification of ChIP-derived samples a calibration curve was made using plasmid pKC201 that carries the entire *ENA1* promoter [11]. In all ChIP experiments, no-template controls, no-IP controls and input samples were tested in parallel for every primer set used. To determine enrichment, the 2^-∆∆Ct^ method was used by comparing enrichment values for positive primer pairs to a negative primer pair between experimental ChIP experiments and reference (pKC201). The amount of *ENA1* mRNA was quantified with a calibration curve made with plasmid pCM262-ENA1-GFP [26] containing the entire *ENA1* DNA sequence. The *ENA1* and *ACT1* (used as a reference) specific pairs of primers (S1 Table) were used to determine the respective mRNA levels.

### β-galactosidase activity assays

The wild type strain BY4741 and its derivative *crz1*::*kanMX4* mutant strain were transformed with plasmid pKC201 [11,33], which contains the entire promoter of *ENA1* fused to *lacZ*. Transformed yeast cells were grown to saturation under selective condition in SD media and then inoculated in YPD at A_600_ 0.2. Growth was resumed until A_600_ = 0.8, then the cultures were distributed into 10 ml aliquots, centrifuged and resuspended in the appropriate media. To induce alkaline stress cells were resuspended in YPD containing 50 mM TAPS adjusted to pH 8.0 with 1 M KOH. Non-induced cells were resuspended in YPD medium. At the appropriate times 1 ml aliquots were centrifuged and β-galactosidase activity was measured as described [34].

### Time-lapse fluorescence microscopy

The time-lapse movie was created by using a combination of microfluidic device and fluorescence microscopy using cells expressing Crz1-3xGFP from plasmid pLMB127. We observed that overexpression had no effect on Crz1 localization dynamics (compared with SP017 cells containing chromosomally-tagged GFP version of Crz1). During microscopy experiments cells were maintained in a fixed position in the CellASIC microfluidic flow chamber (Y04C plate) controlled by the ONIX Control System (CellASIC) and followed by fluorescence microscopy during repeated exchange of medium of pH 5.8 and pH 8.0. The microfluidic device was mounted onto a Nikon TE2000-E inverted microscope with Hamamatsu Camera Controller C-4742-80-12AG, and images were taken with an oil-immersion 40x objective. GFP excitation was detected by illumination with blue light (450–490 nm) and images were acquired at 10 s intervals, automated with Metamorph (Molecular Devices).

For quantification of cytoplasmic vs. nuclear Crz1 in a cell population before and after induction of alkaline pH stress, living cells carrying Crz1-GFP and Sik1-mRFP chromosomally-tagged versions (SP039 strain) were monitored by confocal microscopy over a time course of 20 min. The mRFP signal was used to define the position of nucleus (Sik1-mRFP is constitutively localized in the nucleus / nucleolus). Cell suspensions of strain SP039 were deposited onto a glass bottom dish and allowed to settle. Alkaline pH stress was induced by gentle removing the media placing the pipette tip at the edge of the dish and then quickly replaced by the low-fluorescent medium adjusted to pH 8.0. The Leica TCS SP2 confocal laser scanning microscope equipped with objective HCX PL APO 63x/1.40–0.60 oil was used to obtain confocal images. Images were acquired using emission interval 450–490 nm for GFP and 580–660 nm for mRFP. Pinhole was 1.25 AU. Generated GFP, mRFP, merged GFP-mRFP and bright field images of cell populations at all time points were analyzed using LAS AF Lite (Leica Microsystems) software. Bright field images were taken slightly below (~2 μm) the focal plane until a dark ring surrounds cell (useful for the Cell-ID software to find boundaries of cells, see below).

### Image analysis

We performed image analysis and data processing using the open-source software packages ImageJ and Cell-ID version 1.4 as described [35]. Briefly, a time-related series of GFP, mRFP and out-of-focus bright field images acquired by confocal microscopy were converted into grey-level TIFF files of 8 bits using ImageJ software. Individual cells of regular yeast shape were found by Cell-ID software using an algorithm that calculates cell boundaries based on comparison of pixels of dark cell boundaries and bright background. The cell boundaries were then translated to the corresponding fluorescence images (from both GFP and RFP channels) and the values corresponding to either nucleus, total or background fluorescence were calculated. To calculate the nuclear fluorescence signal of Crz1-GFP in a single cell, we divided the nuclear fluorescence value by the calculated nuclear area and subtracted fluorescence background signal. The final nuclear GFP value was corrected for photobleaching effect as follows: the region of interest (ROI) of 65 individual cells was bleached with a 488 nm laser under the same conditions as the experiment was performed (i.e., scan speed, zoom, laser power, number of exposures to laser, microscope objective). Average fluorescence intensity of all ROIs for each time point was calculated and normalized to initial fluorescence (100%). The intensity drop attributable to the bleaching of a single ROI was typically 20% at the end of the experiment. Calculated average intensity loss per each time point was subtracted from the nuclear Crz1-GFP values of the alkaline pH experiment. In total approximately 600 individual cells exposed to high pH were tracked.

### Other techniques

Cell volume of BY4741 cells growing in YPD supplemented with 50 mM TAPS before and 2 min after induction of alkaline pH stress was measured using a ScepterTM 2.0 Cell Counter and 40 μm-Scepter Sensors (Millipore) according to the manufacturer’s instructions.

### Mathematical modeling

The mathematical models created here use systems of ordinary differential equations. Given that the mechanisms involved in the various processes are not known in detail, we used the power law formalism [36–38]. This formalism allows us to mathematically represent linear and non-linear dynamics of biological processes whose details are poorly characterized [39,40]. This formalism facilitates the systemic integration of processes into a single mathematical model, enabling the analysis and understanding of the integrated behavior in the system (see Methods and [41–44]. The power law formalism has two types of parameters: apparent rate constants and apparent kinetic orders.

We used constrained optimization to estimate parameter values for the models. The constraints are used as a means to ensure that parameter values have physically plausible values. Apparent rate constants are constrained to be larger than zero, as negative rate constants are physically impossible. Apparent kinetic orders are constrained to be larger than zero and smaller than a small integer, as described [45]. This is due to the fact that such kinetic order cannot be larger than the number of binding sites for the variable in the process of interest.

Differential equations were fitted to the experimental data as described in [45,46]. We implemented the parameter fitting procedure using the NonLinearModelFit function in Mathematica, for all parameter fitting calculations. Initial estimates for the parameter values in the fitting process were obtained as described in S1 Text.

A commonly accepted general assumption was made in the modeling of mRNA dynamics: we assumed that mRNA degradation linearly depends on mRNA abundance. This assumption has been used to model mRNA degradation in many systems, ranging from bacteria, to multicellular eukaryotes (e.g. [45,47–53], and including yeast (implicit in Eq. 6 of ref. [54] and explicit in Ref. [55]). Although simplifying, this assumption is sufficiently accurate in most cases (e. g. [45,49,51] and is consistent with many experimental measurements (e.g. [53,54]).

## Results and Discussion

### Time-course analysis of *ENA1* promoter activity and mRNA accumulation

**Fig 1:**
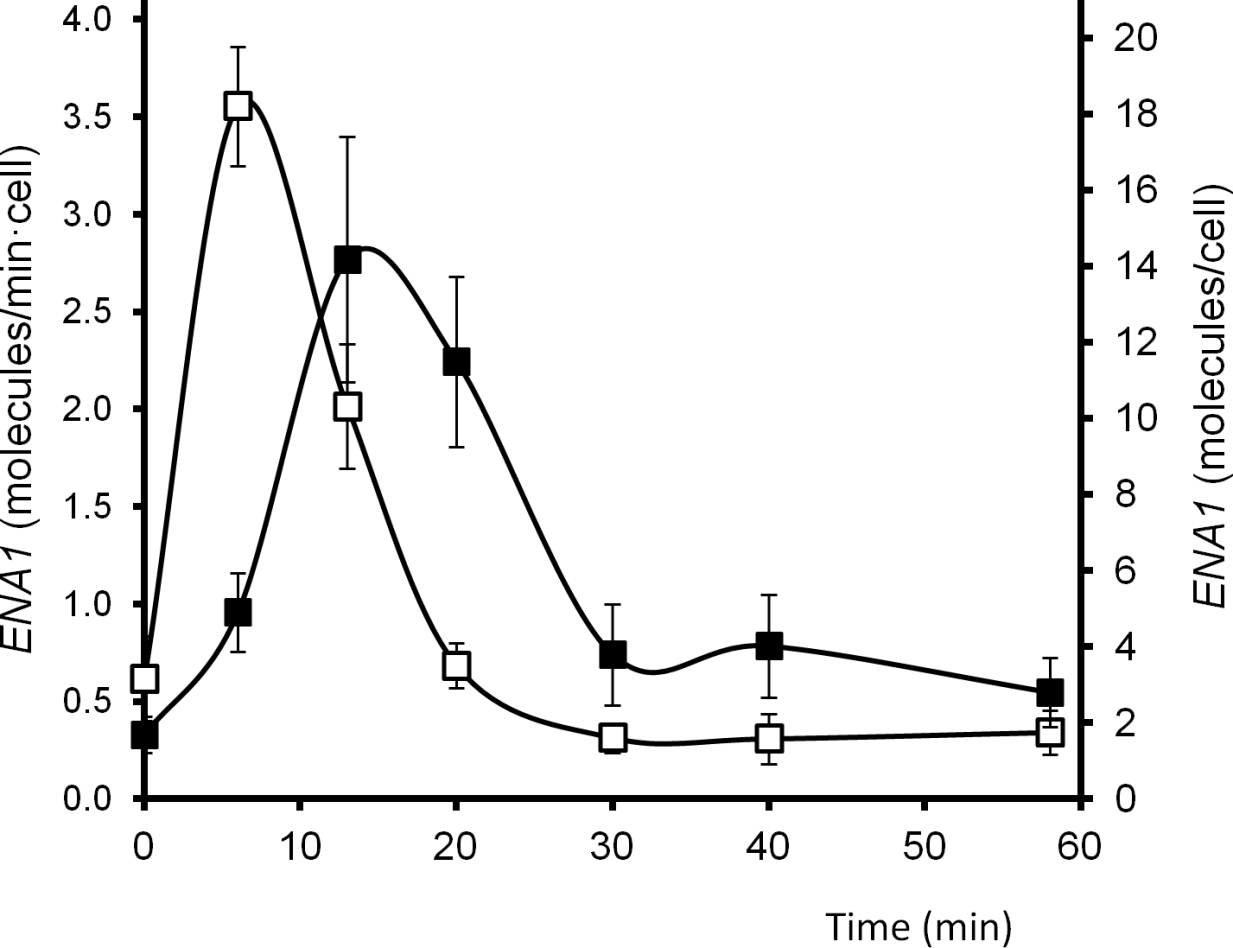
Transcription rate (TR) and mRNA accumulation (RA) of *ENA1* upon high pH stimulation. The transcription rate (open squares) and mRNA amount (closed squares) for the *ENA1* gene under alkaline stress was obtained from GRO experiments [27]. In order to convert the radioactive intensities into real units, the mRNA concentrations and mRNA half-lives for the most expressed genes published in (Wang *et al*., 2002) were compared with radioactive intensities of RA and TR for the same genes at time 0 (steady-state conditions) to obtain a conversion factor. The conversion factor was applied to the complete set of GRO data under alkaline stress. *ENA1* data were extracted from this set and represented as the average ± SEM of three experiments.

The promoter activity of *ENA1* was calculated from the data recently obtained from our Genomic Run On (GRO) analysis [27] of the changes caused by sudden transition from pH 5.5 to a moderate alkalinization (pH 8.0). This specific shift was selected because it generates a vigorous *ENA1* mRNA accumulation response and results only in a transient delay in proliferation with no significant loss of cell viability. In addition, we determined that this change barely affects cell volume (60 fL at pH 5.5 *vs* 56 fL at pH 8.0). As shown in Fig 1, transcription of *ENA1* peaks after 6 min of initiation of the stress and at this moment the transcription rate is estimated in 3.5 molecules/min*cell. *ENA1* mRNA levels peaks at 13 min after the stress, and reach a maximum of 14 molecules per cell. Therefore, the transition between peak transcription and maximum mRNA levels takes approximately 7 min. The comparison of the profiles of transcription rate and mRNA levels suggests that changes in mRNA stability would play a relatively minor role and that the amount of *ENA1* mRNA would depend largely of the activity of the promoter.

### Kinetics of alkaline pH-induced nuclear-cytoplasmic shuttling of Crz1

**Fig 2:**
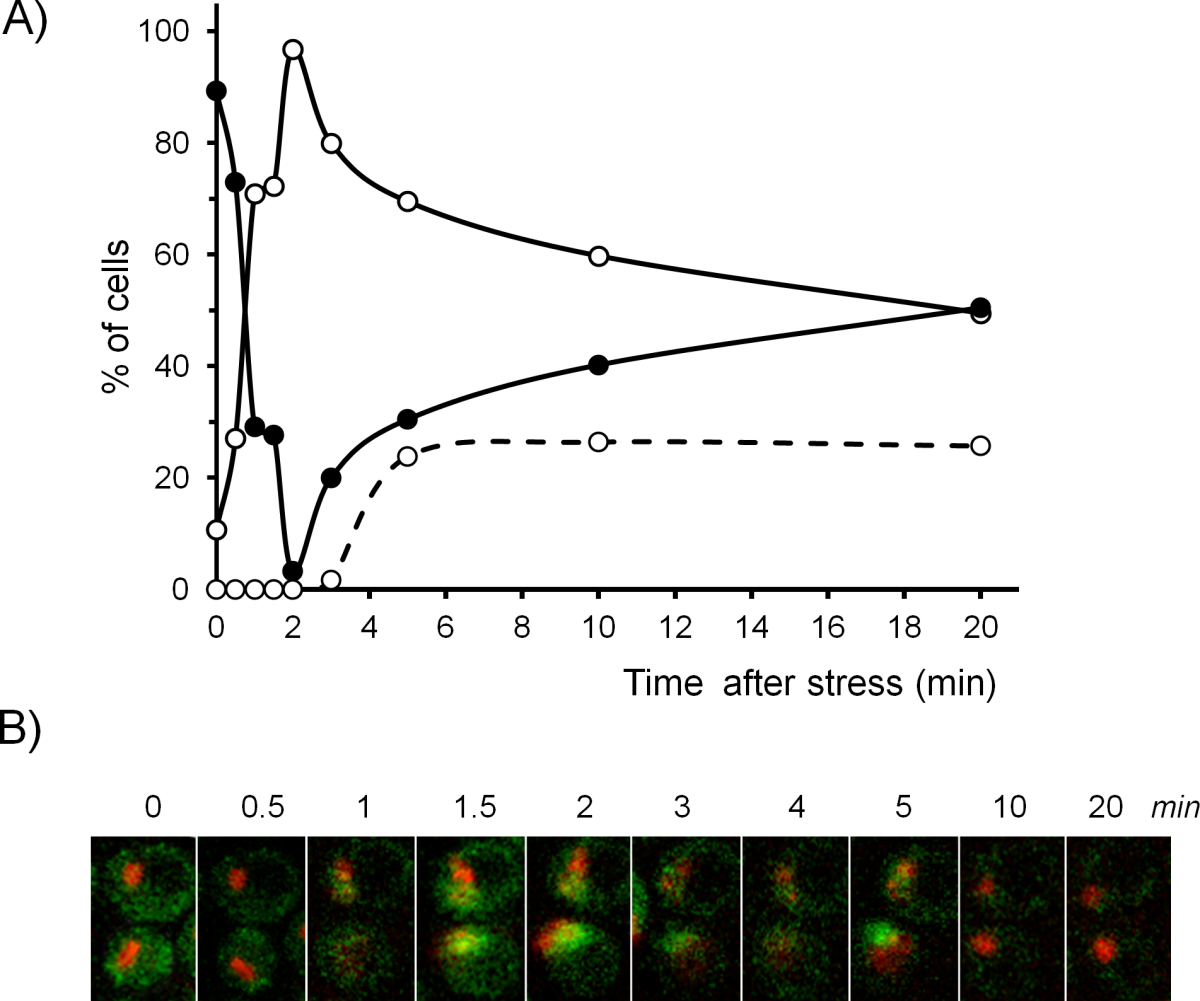
Time-course of the subcellular distribution of Crz1 upon high pH stress. A) Cultures of strain SP039 (*CRZ1*:GFP-*kanMX6 SIK1*:mRFP-*kanMX6*) were shifted from pH 5.5 to pH 8.0 and the localization of Crz1 monitored by fluorescence confocal microscopy as described in Materials and Methods. Open circles denote the percentage of cells with nuclear Crz1 localization; closed circles, cytosolic localization. The discontinuous line show cells in which Crz1 entered the nucleus, then left the nucleus and later returned to it (they are, therefore, a subset of the total nuclear Crz1 values). Sik1 signal was used as marker of constitutive nuclear (nucleolar) localization. Data is presented as percentages calculated for 90 to 160 cells examined for each time-point. B) Example of time-lapse images of SP039 cells before and after induction of alkaline stress over a 20 min time-course (as in panel A).

The rapid response of the *ENA1* promoter is congruent with a substantial presence of the calcineurin-activated transcription factor Crz1 in the nucleus after 2.5–5 min upon alkaline stress, as it was documented earlier [17]. We therefore considered necessary to quantitatively monitor, in a time-resolved manner, the traffic between cytosol and nucleus of Crz1 upon alkalinization. To this end, we used strain SP039, which expresses chromosomally encoded version of Crz1 fused to GFP and Sik1 (a nuclear localization marker) fused to mRFP. The subcellular distribution of Crz1 in cells shifted to pH 8.0 was monitored using time-lapse confocal microscopy during 20 min. As observed in Fig 2A, an increase in the number of cells with nuclear Crz1 localization is already detected after 30 s of alkalinization, and a peak with >95% of cells show nuclear localization of the transcription factor, is reached after 2 min. Interestingly, the subsequent rapid decline in the number of cells in which Crz1 is nuclear is attenuated after 5 min of the onset of the stress due to a second wave of Crz1 returning to the nucleus that is observed in about 25% of the cells monitored (an example is shown in Fig 2B). Although in a given cell Crz1 could subsequently shift back again to the cytoplasm, the overall percentage remained constant during the rest of the experiment, with about half of the cell population showing nuclear Crz1 localization. The extremely fast nuclear entry of Crz1 shown in Fig 2A is in keeping with the almost immediate peak of intracellular calcium detected 10–20 sec after raising the pH of the medium from 5.5 to 8.1 [15] and suggests that the interaction of Crz1 with the *ENA1* promoter is an important determinant in the rapid transcriptional induction observed.

It is worth noting that the pattern of nuclear accumulation of Crz1 upon high pH stress differs from previously reported examples. Thus, it has been documented that significant accumulation of Crz1 in the nucleus upon exposure to calcium occurs only after 5 to 20 min [21,56–58], whereas 10 to 15 min of incubation with some toxic compounds, such as methylglyoxal or arsenite, is required to detect nuclear Crz1. For other stimuli able to activate calcineurin, such as blue light, a period of as much as 10–20 min is necessary to detect significant nuclear accumulation of Crz1 [59]. This is in contrast with the very rapid accumulation of Crz1 in the nucleus upon alkaline stress. In addition, treatment of yeast cells with 0.2 M calcium results in sporadic, unsynchronized nuclear localization bursts [57], whereas nuclear localization of Crz1 upon exposure to blue light is persistent [59]. It must be noted that the second wave of nuclear Crz1 detected in our experiments cannot be explained by the nuclear localization bursts observed upon stimulation of the cells with calcium [57], since such bursts typically lasted 2 min, whereas we observe a rather stable profile during the time of alkaline stress (see S1 Video in Supplemental Material). Therefore, alkaline stress might invoke a distinctive and unique pattern of nuclear accumulation of Crz1. It is currently unclear whether this late return of Crz1 to the nucleus is caused by a second spike of cytosolic calcium, because after the initial burst upon alkaline stress, calcium levels return to baseline values in a matter of seconds and remain unchanged for at least three minutes [60]. It is worth noting that nuclear Crz1 regained cytosolic localization very shortly (1–2 min) after extracellular pH decreased to pH 5.8 and returned quickly to the nucleus when pH was raised again to 8.0, with no signal of refractory period (see accompanying S1 Video).

**Fig 3:**
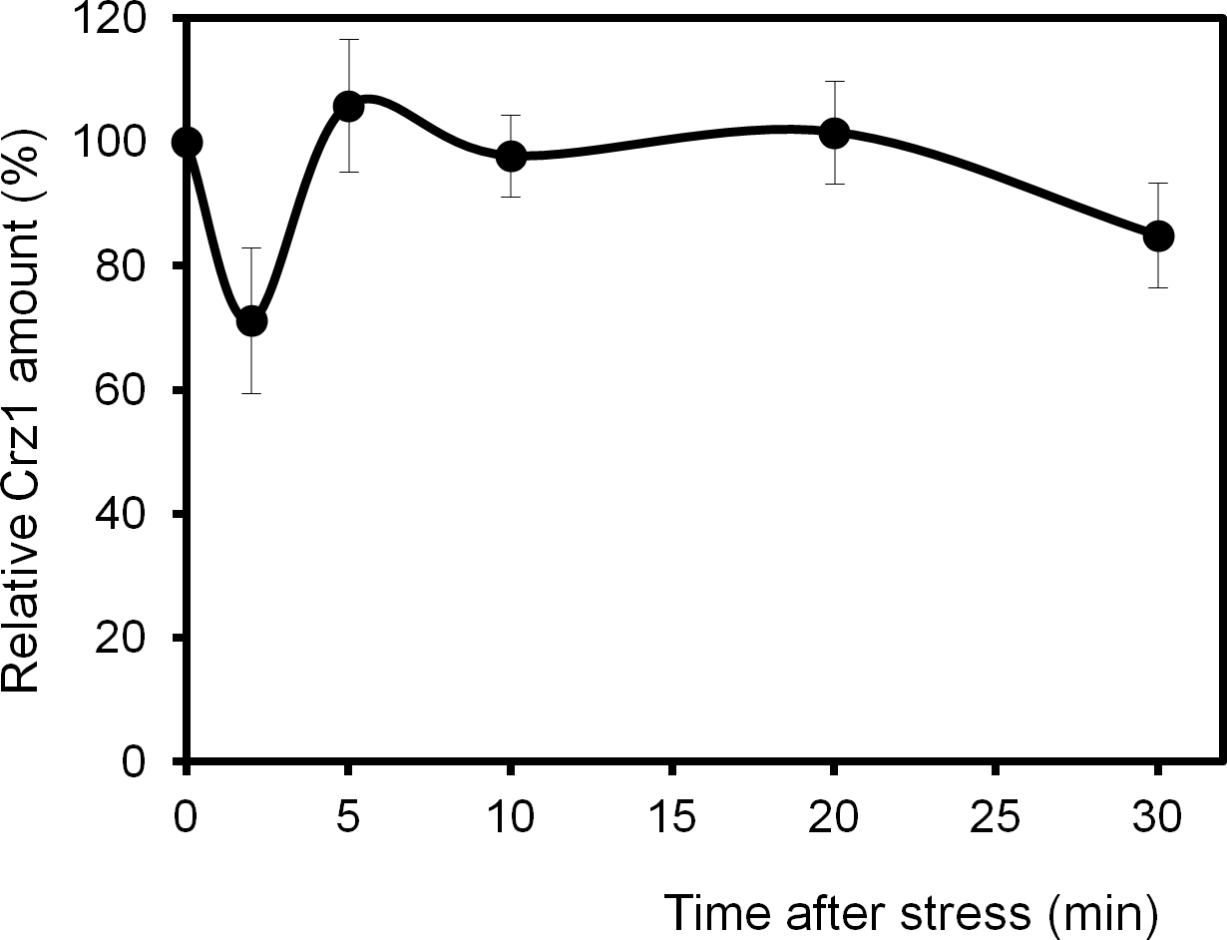
Relative levels of Crz1 after switching cells to pH 8.0. The number of Crz1 molecules/cell was calculated as described in Material and Methods and the value just prior alkalinization of the medium was set as the reference (100%). Data correspond to the mean ± SEM from 8 independent experiments.

**Fig 4:**
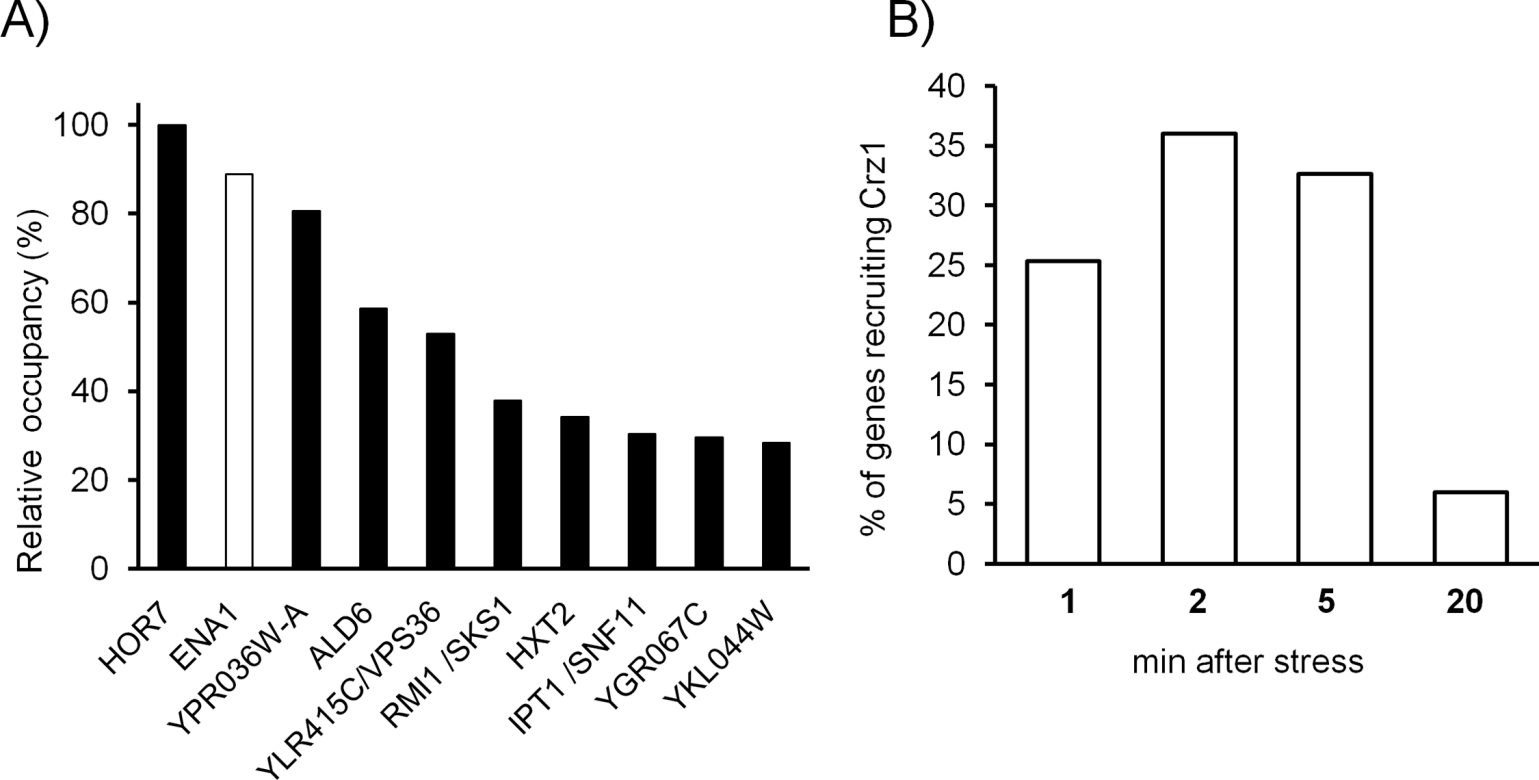
Genome-wide recruitment of Crz1 to gene promoters in response to high pH stress. ChIP-seq technology was used to identify intergenic regions immunoprecitating with Crz1 and accumulating at least two-fold reads than the genome average. A) The 150 genes showing peaks of reads above the indicated threshold were ranked according the number of reads at the peak. The relative occupancy with respect the highest value (*HOR7*) is shown for the top ten genes in the ranking. B) The percentage of genes showing the peak of accumulation of reads at a given time after alkalinization of the medium is shown.

Although significant changes in *CRZ1* gene expression upon cell stress have not been reported, we considered important to monitor, in addition to its subcellular localization, hypothetic changes in the cellular amounts of the transcription factor after high pH stress. This was tested by immunoblot using a strain expressing a chromosomally encoded Crz1 protein C-terminally tagged with a triple HA epitope and using known amounts of recombinant GST-3HA-Ypi1 protein as an internal standard for signal comparison. Using this method we determined a value of 186 ± 32 molecules of Crz1/cell in non-stressed cells, which is somewhat lower than that reported by most previous determinations [60]. It must be noted that the existing measurements largely differ among them (up to 20-fold). In any case, the amount of Crz1 during the first 30 min upon high pH stress did not change significantly (Fig 3), with the exception of the minute time point, 2 where a decrease of almost 30% was observed (*p*<0.05). However, we believe that these variations occur too fast to represent actual changes in the amounts of cellular Crz1 and we postulate that this difference, which corresponds to the time of maximum nuclear Crz1 accumulation (see Fig 2A) and massive Crz1 binding to its genomic target regions (Fig 4B), might be an artifact caused by the inability of the method used for protein extract preparation to quantitatively recover nuclear, DNA-bound Crz1.

### Analysis of Crz1 binding to the *ENA1* promoter upon high pH stress

As a next step, we studied the kinetics of Crz1 binding to the *ENA1* promoter after high pH stress. This was done by genome-wide examination of Crz1 binding to high pH-responsive promoters by ChIP-Seq analysis followed by specific analysis of previously identified CDREs. Samples were taken 1, 2, 5 and 20 min after shifting cells from pH 5.5 to 8.0. Accumulation of Crz1 was detected at 152 intergenic regions, of which 100 could be attributed to specific genes, whereas 52 corresponded to divergent promoters. *ENA1* showed a very robust response, as it was observed to be the gene with the second highest number of reads at the promoter (Fig 4A). No reads were mapped at the *ENA2* and *ENA5* promoter regions, in agreement with the almost null response of these genes to alkaline stress (data not shown).

Recruitment of Crz1 was a very rapid process (Fig 4B), with over 60% of the promoters showing maximum binding within the first two minutes after alkaline pH stress. In many cases (66%), binding to the promoter decreased substantially after 20 min (as determined by the % of cases in which reads at that time were less than at any other time after the onset of stress). These observations fit well with the timing for nuclear Crz1 entry described above (see Fig 2). A detailed study of the genome-wide promoter binding of Crz1 will be published elsewhere (Roque *et al*. manuscript submitted).

Concerning *ENA1*, maximal occupancy was observed at 2 and 5 min after alkalinization, with significant increase already after one min. Interestingly, occupancy of the promoter region was still substantial (∼65% of the maximum) after 20 min, suggesting a sustained activation. This is in contrast with the very low transcription rate observed at 20 min (Fig 1), and suggests that negative regulatory components of *ENA1* transcription that are removed from the promoter upon high pH stress are resuming their repressor function. This interpretation fits with the observation that the Mig2 repressor, known to act on the *ENA1* promoter [14], exits the nucleus in the first few minutes upon alkalinization but recover their nuclear localization 15 min after induction of the stress [5].

**Fig 5:**
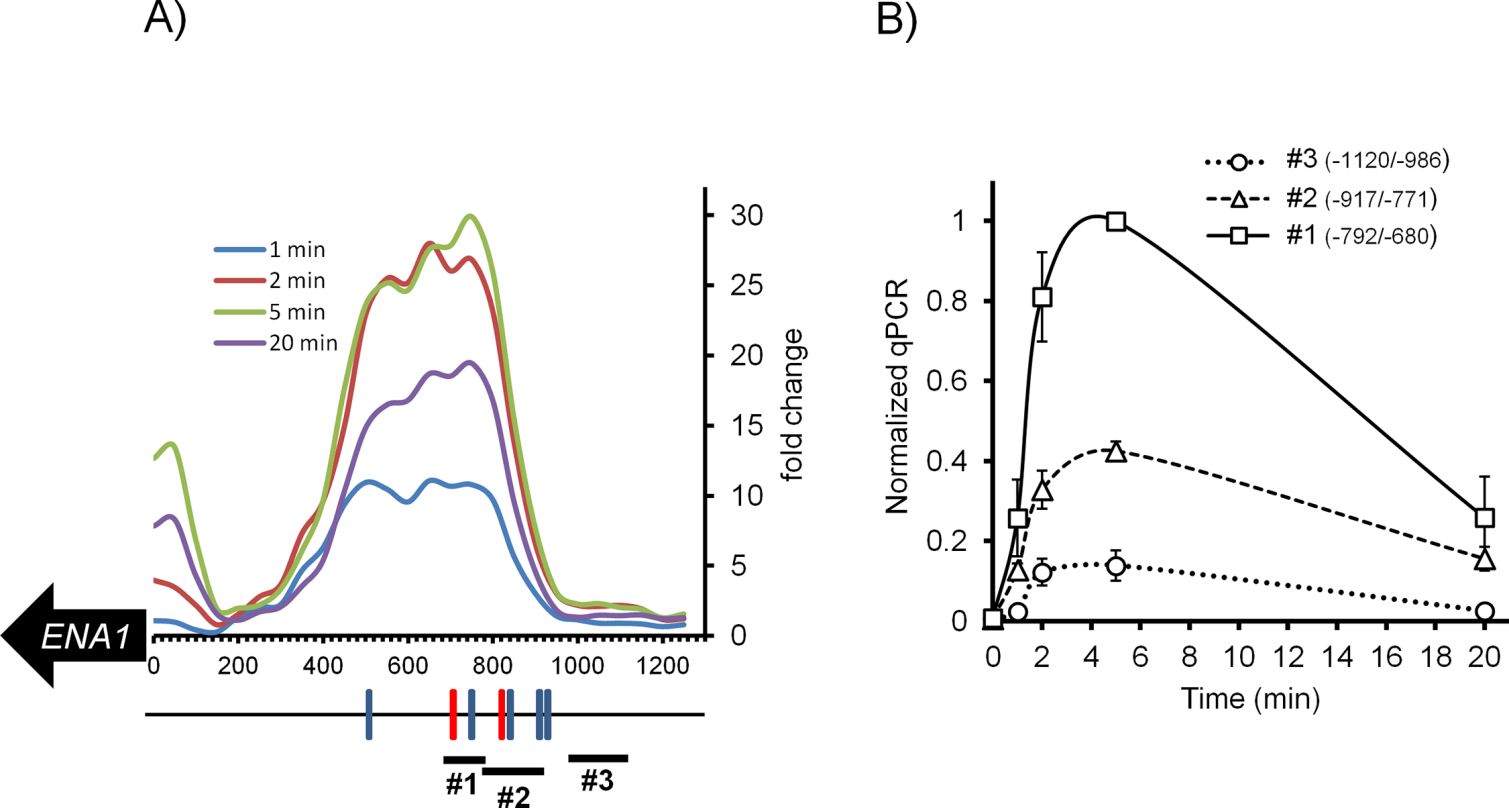
Time-course profile of Crz1 recruitment at the *ENA1* promoter following alkaline stress. A) Cumulative counts of reads using a 50-nt window spanning the *ENA1* promoter region are represented as fold-change over reads counted at time 0. The positions of CDRE deduced from computational analysis using the PWMTools website (http://ccg.vital-it.ch/pwmtools/pwmscan.php) of the Swiss Bioinformatics Institute are indicated as colored boxes on the black line. Red boxes denote the CDRE previously characterized in [23]. B) Evaluation of relative Crz1 binding to CDREs at positions -813/-821 and -719/-727 (red boxes in panel A). Chromatin immunoprecipitation using HA-tagged Crz1 was carried out and the immunoprecipitate used to amplify the -680/792; -771/-917; and -986/-1120 promoter regions (denoted as thick short lines 1, 2 and 3, respectively, in panel A). Data is presented as the mean ± SEM from 3–4 individual experiments assayed in duplicate and expressed as relative to the maximum value.

As shown in Fig 5A, several putative CDREs can be predicted in the *ENA1* promoter, although the accumulation of Crz1 peaks in a region containing the two CDRE regions characterized by Serrano's laboratory [23], denoted in red in Fig 5A. Because of the close proximity of both CDREs (-813/-821 and -719/-727 relative to the *ENA1* initiating Met codon) ChIP-Seq data does not provide the required resolution to evaluate the relative contribution of each element for Crz1 binding to the promoter. To solve this question we carried out ChIP experiments followed by qRT-PCR analysis using oligonucleotide pairs specifically encompassing each CDRE (denoted as #1 and #2 in Fig 5A). As shown in Fig 5B, the downstream element (-719/-727) was more effective in recruiting Crz1 (>2-fold compared with the upstream one). In both cases binding was much more prominent after 2 and 5 min of shifting cells to pH 8.0. Amplification of a region between nt -986/-1120 (#3), for which no CDRE is predicted, showed only residual accumulation of Crz1. These results confirm *in vivo* the previously observed *in vitro* binding of Crz1 to these sequences [23], although our data suggest that both CDREs are stress-responsive (see below). Recruitment to the region -493/-595, containing the putative CDRE at position -528/-537, was barely above the negative reference region (not shown), suggesting that this sequence is not functional. This perfectly fits with the existing evidence that the region between nucleotides -573/-490 of the *ENA1* promoter was unable to drive transcription when cells were exposed to calcium chloride and that, while able to transcriptionally respond to high pH, this effect was insensitive to FK506 (an inhibitor of calcineurin) and not affected by mutation of *CRZ1* or *CNB1* [10].

### Contribution of Crz1 to alkaline pH-induced *ENA1* transcription and protein accumulation

The contribution of Crz1 to the increase of *ENA1* mRNA levels was investigated by qRT-PCR. As shown in Fig 5A, mRNA levels in the wild type strain peaked at 15 min with a value of approximately 25 molecules/cell. These kinetics profile of accumulation fits fairly well with that shown in Fig 1, taking into account the different technologies employed (Fig 1 data derives from DNA macroarray experiments [27]. Lack of Crz1 results in a drastic reduction of mRNA accumulation (∼ 6 molecules/cell). In addition, the peak is observed slightly earlier than in wild type cells (10 min, instead of 15). This indicates that Crz1 is not the only factor triggering the early induction of the *ENA1* promoter.

**Fig 6:**
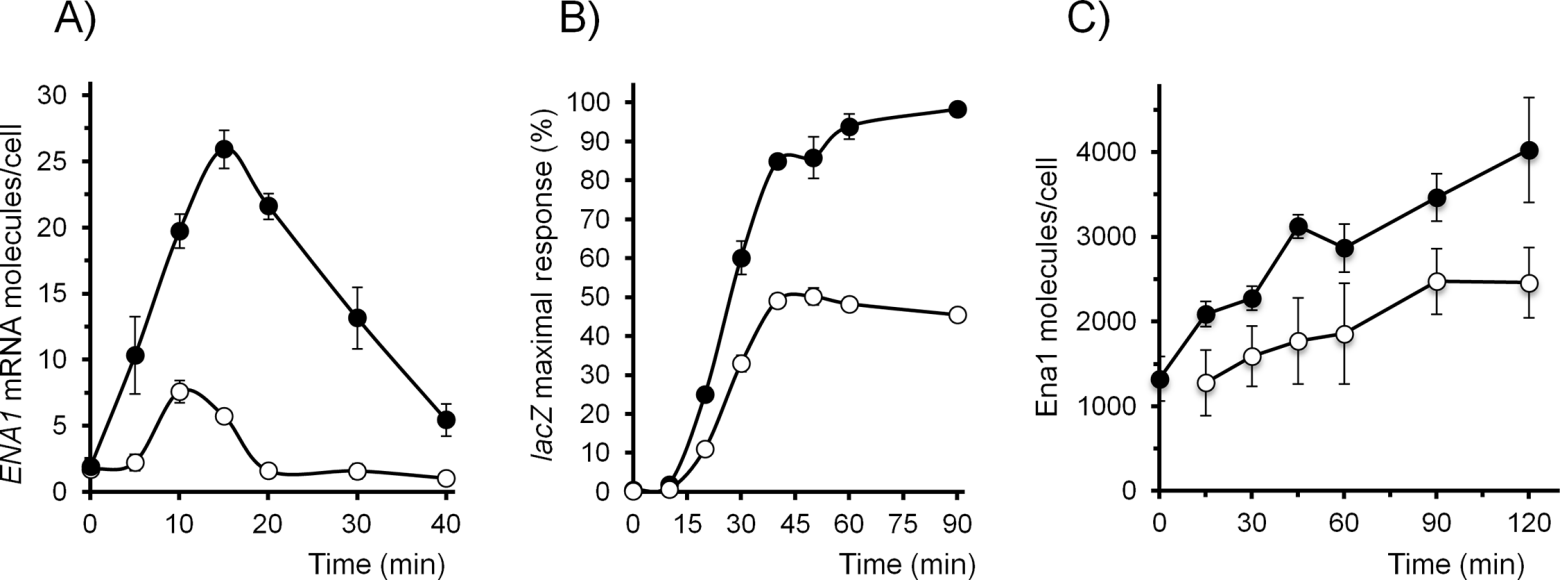
Impact of the absence of Crz1 on mRNA levels and Ena1 protein accumulation after high pH stress. A) Time-course of the accumulation of Ena1 mRNA after shifting cells to pH 8.0. Total RNA from wild type (closed circles) and *crz1* (open circles) cells was extracted and mRNA levels were determined by qRT-PCR and quantified as indicated in Materials and Methods. B) The strains described above were transformed with plasmid pKC201, carrying the *lacZ* reporter fused to the *ENA1* promoter. Cells were exposed to pH 8.0 for the indicated times, collected, and β-galactosidase activity measured. C) Strain MLM001 (*CRZ1*) and MLM002 (*crz1*), carrying in both cases C-terminally GFP-tagged version of *ENA1* were shifted to pH 8.0 for the indicated times and the amount of total Ena1 quantified by immunoblot using anti-GFP antibodies and internal standards of recombinant GFP protein. Data is presented as the mean ± SEM from 3 (panel A) or 4 (panels B and C) independent experiments.

We then tested the effect of the absence of Crz1 on the *ENA1* promoter using a translational fusion of this promoter with the *lacZ* gene, encoding β-galactosidase. In contrast to the dramatic effect of the absence of Crz1 on *ENA1* mRNA levels, a reduction of only about 50% of the total activity was detected (Fig 6B). We next monitored by immunoblot the total amount of Ena1 protein produced in the cell using a GFP-fused version of the protein. As shown in Fig 6C, the abundance of Ena1 in non-stressed cells was estimated in 1324 molecules/cell. This value is very close to the estimated value integrated from the existing literature for non-stressed cells [60] although it must be noted that the experimentally reported values show a very wide range (from nearly absent to about 7000 molecules/cell). Stimulation of cells with pH 8.0 caused the almost continuous accumulation of Ena1, reaching 4030 molecules/cell at the end of the experiment (120 min). Evaluation of Ena1 in the *crz1* mutant background in non-stressed cells was not possible because the amount of the protein was below the sensitivity of the assay (∼500 molecules/cell). Upon induction by high pH, we observed that Ena1 accumulated at lower rate than in wild type cells, reaching a maximum (2476 molecules/cell) at 90 min.

### Data-driven modeling of Crz1-regulated Ena1 dynamics

The experimental results described above were integrated into a multilevel mathematical model in order to understand the differences between the dynamic behavior of Ena1 in wild type and mutant strains from 0 to 60 min after the stress challenge. We modeled the dynamic behavior of Ena1 at the mRNA and protein levels, through a set of ordinary differential equations that use the power law formalism (see Methods).

Eq 1 describes the dynamic behavior of *ENA1* mRNA:

mRNA'=α0(t)+α1Crz1g1-α2Ena1mRNA Eq 1

**Fig 7:**
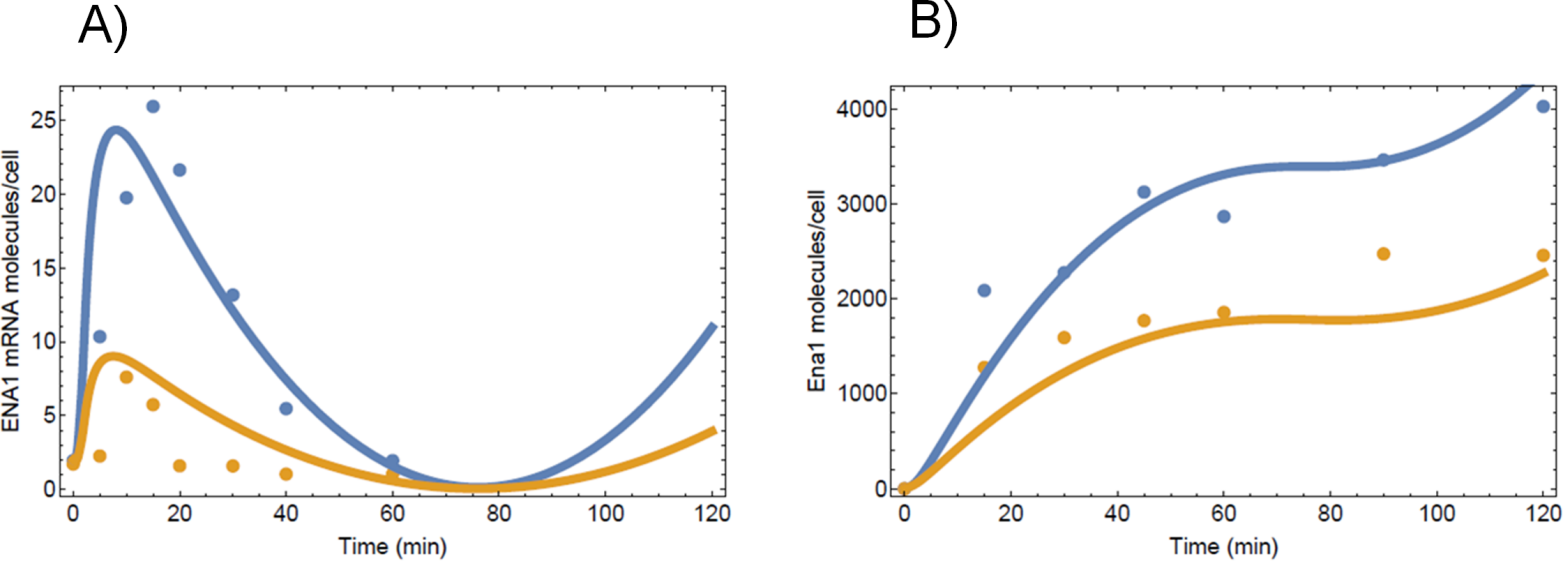
Modeling of Ena1 mRNA and protein abundances upon high-pH stimulation. Time-course of the Ena1 mRNA (panel A) and protein accumulation (panel B) after shifting cells to pH 8.0. Experimental data are shown as dots and the mathematical estimations, as lines (blue for wild type and yellow for mutant strains).

As observed in Fig 7A, there is a basal expression of Ena1 mRNA that is independent of Crz1. This is represented by the term α0(t), which we estimated using a time-dependent spline function adjusted to fit the curve corresponding to the *crz1* mutant in Fig 6A. The term α1 Crz1n^g1^ represents Crz1 driven Ena1 mRNA synthesis. α1 represents an apparent rate constant, Crz1 represents the nuclear Crz1 concentration, and g1 represents the apparent kinetic order of nuclear Crz1 in the Ena1 mRNA synthesis. Values for α1 (= 0.00025 molecules/cell min^-1^) and g1 (= 2) were obtained by fitting this term to a time curve obtained by subtracting Crz1-independent Ena1 mRNA synthesis from total Ena1 mRNA synthesis (*crz1* curve—WT curve in Fig 6A). Finally, the term α2 Ena1_mRNA_ represents the degradation of Ena1 mRNA. The value for the apparent rate constant α2 was calculated after assuming that degradation linearly depends on mRNA concentrations (see **Methods**). Using the parameter fitting procedure described in Methods and the data from Fig 7A we estimated that α2 = 0.2 molecules/cell min^-1^).

Eq 2 describes the dynamic behavior of the Ena1 protein:

Ena1pr'=α3Ena1mRNAg3-α4Ena1pr Eq 2

The term α3 mRNA^g3^ represents protein synthesis. We calculate the values for α3 (= 14 molecules/cell min^-1^) and g3(0.6) by adjusting this function to to experimental data (Fig 7B). The term α4 Ena1pr represents the degradation of the Ena1 protein. This degradation is also assumed to be proportional to the amount of protein, as it has been reported to occur in many situations [54]. The rate constant α4 (= 0.3 molecules/cell min^-1^) is also calculated by fitting the function to the *crz1* experimental data from Fig 6C.

The parameter values given above were calculated for the wild type strain. For the *crz1* mutant strain, Ena1 mRNA dynamics is represented by Eq 3

mRNA'=α0(t)-α2Ena1mRNA Eq 3

We maintain the values calculated for the wild type α0(t) and α2 in the mutant model. The dynamic behavior of the wild type and *crz1* models are given in Fig 7, where we also show the experimental dynamic behavior of the system. As observed, these models fit the experimental data very well, as shown by the Adjusted R^2^ (0.72 for the mRNA and 0.87 for the protein). These values indicate the percentage of variation in the experimental measurements that are justified by the models. In addition to the well adjusted R^2^s of our models, there is another experimental observation that supports our model. Based on the available experimental data, our parameter fitting for the multi-level model only uses data from times 0 to 60 min after stress induction. Nevertheless, Ena1 protein level measurements are available for up to 120 minutes after stress induction. When we run the model for 120 minutes, it quantitatively describes the full 120 min time course for Ena1 (Fig 7).

### Model-based biological hypothesis generation

There are several hypotheses that can be inferred from this data driven model. The first hypothesis relates to the function of Crz1 in regulating *ENA1* mRNA gene expression. Crz1 relocates from the cytoplasm to the nucleus during the first stage of the stress response. It then shuttles back to the cytoplasm, and subsequently part of the Crz1 population returns to the nucleus. It must be noted that correct fitting of our model to the observed dynamic behavior of the wild type and *crz1* strains requires the second wave of nuclear Crz1 to be fully effective in regulating *ENA1* gene expression. The second hypothesis comes from that fact that the value for the apparent kinetic order for Crz1 in the Crz1-dependent Ena1 mRNA synthesis is 2, which would be consistent with two effective Crz1 binding sites required for Ena1 mRNA synthesis. This would fit with our *in vivo* data (Figs 6 and 7) and previous evidence indicating *in vitro* binding of Crz1 at two separate promoter regions [23]. It has been shown that mutation of the downstream *ENA1* CDRE element was able to largely abolish the transcriptional response to calcium and high pH stress, and there is evidence that short regions containing the *ENA1*–712/-733 region are able to drive transcription in response to calcium [23], high pH [10], or as a result of the deletion of the *PPZ1* phosphatase gene [61]. Therefore, it seems clear that *in vivo* binding of Crz1 to this genomic region triggers a functional transcriptional response resulting from activation of calcineurin. In contrast, it was shown that the upstream element alone was unable to drive transcriptional response to calcium stress, leading to the proposal that this region was mostly responsible for basal expression of *ENA1* [23]. However, we find significant recruitment of Crz1 to this region in response to high pH stress, accounting for about one third of the total recruitment to the promoter (Fig 5B) and our model supports a 2-site regulatory scenario. It is plausible that recruitment of Crz1 to the downstream region alone would be insufficient to activate the promoter, but would enhance the effect derived from Crz1 recruitment to the upstream region. Such synergistic effect would fit with the model proposed earlier by Mendizabal and coworkers [23].

Our third hypothesis is somewhat more complex. We observe that the time course for the stress-dependent expression of Ena1 mRNA in the *crz1* mutant almost perfectly correlates with that of the wild type. This can be seen when calculating the correlation between β-galactosidase activity driven by the *ENA1* promoter in wild type and *crz1* strains (Spearman correlation = 0.83). This high correlation suggests that Ena1 mRNA expression might be regulated by other transcription factors that have a dynamic behavior similar to that of Crz1. If this is so, the dynamics of Crz1 in the wild type could be used as a proxy to estimate the dynamics of these other transcription factors in the *crz1* mutant, allowing us to rewrite Eq 3 as:

mRNA'=f(Crz1(t)wt)-α2Ena1mRNA Eq 4

If we approximate f(Crz1) using the same power law formalism as above, then

mRNA'=α'0Crz1wtg0'-α2Ena1mRNA Eq 5

Adjusting this new function to the experimental data, we obtain α'0 = 0.00015 and g0' = 2. We note that α'0≈α1/1.7, which is the approximate average ratio between the expression of β-galactosidase from the *ENA1* promoter in the wild type vs. the *crz1* mutant. This suggests that the regulation of Ena1 mRNA expression by its different transcription factors during stress response might be additive. In addition, the value for g0' suggests that the number of effective binding sites for transcription factors inducing stress-dependent Ena1 mRNA expression is 2. However, as deduced from Fig 6A, this Crz1-independent event must take place very shortly after the initiation of the stress. It is known that activation of *ENA1* upon high pH stress is under the direct control of three pathways: calcineurin/Crz1, Snf1 and Rim101 [14],. However, the Rim101 pathway affects *ENA1* expression by repressing the expression of the Nrg1 repressor [62] which binds to the *ENA1* promoter at a region located between nucleotides -561/-573 [14]. Therefore, the impact of the activation of Rim101 on *ENA1* transcription can only occur at medium-long term time-points. In fact, a delayed effect for Rim101 action has been shown for the high-pH responsive *PHO89* promoter [5]. In contrast, Snf1 is rapidly activated by phosphorylation in response to high pH stress [5,63]. Snf1 can functionally interact with two pairs of repressors, Nrg1/Nrg2, and Mig1/Mig2. Nrg1 has been proposed to mediate Snf1-activated function in response to some stress conditions [64–66] and very fast Mig1 and Mig2 Snf1-dependent phosphorylation, concurrent with rapid exit of these repressors from the nucleus, has been recently documented [5]. As both Nrg1 and Mig2 are known to repress the *ENA1* promoter under non-stress conditions, we propose that the short-term, Crz1-independent *ENA1* transcriptional activity is essentially promoted by activation of the Snf1 pathway.

## Supporting Information

S1 Table (DOCX)

S1 Text (DOCX)

S1 Video Additional Supplemental Material (MP4)
