# Supplementary material for: Regulation of the Na+/K+-ATPase Ena1 Expression by Calcineurin/Crz1 under High pH Stress: A Quantitative Study
Source: PLoS One. 2016 Jun 30;11(6):e0158424. doi: 10.1371/journal.pone.0158424 (PMC4928930; doi:10.1371/journal.pone.0158424)
Supplement: S1 Table — (DOCX) [file pone.0158424.s001.docx]

Supplemental Table 1. Oligonucleotides used in this work

| **Name** | **Sequence** | **Description** |
| --- | --- | --- |
| Left_Ena1_3 | AACAGTGACGATAAGAAACG | PCR of *ENA1* promoter from -1120 to -986 |
| Right_Ena1_3 | CCCTAAAATATTGACCACAC | PCR of *ENA1* promoter from -1120 to -986 |
| Left_Ena1_5 | CTATCTCTAGCCGTTCATCA | PCR of *ENA1* promoter from -917 to -771 |
| Right_Ena1_5 | CAACTTCCGTACAAGAGTGA | PCR of *ENA1* promoter from -917 to -771 |
| Left_Ena1_6 | TCACTCTTGTACGGAAGTTG | PCR of ENA1 promoter from -792 to -680 |
| Right_Ena1_6 | ATCATAACAGGAAGCGATAA | PCR of ENA1 promoter from -792 to -680 |
| CRZ1-C-pFA6-dir | TCACTCCCTTGTACGAAGAAGCCAGACAGGAGAAATCGGGACAAGAGAGTCGGATCCCCGGGTTAATTAA | Construction of strain with chromosomally GFP-tagged *CRZ1* locus |
| CRZ1-C-pFA6-rev | TTATATAGAAAAAAAAAATTCCTATTCAAAGCTTAAAAAAACAAAAATAAGAATTCGAGCTCGTTTAAAC | Construction of strain with chromosomally GFP-tagged *CRZ1* locus |
| ENA1-C-pFA6-dir | TACTACAATCCATACAGAAGTTAATATTGGTATTAAACAACGGATCCCCGGGTTAATTAA | Construction of strain with chromosomally GFP-tagged *ENA1* locus |
| ENA1-C-pFA6-rev | TGAATAAGGAAAAAGATAGGGAGCACTTAATAGGCCCTGCGAATTCGAGCTCGTTTAAAC | Construction of strain with chromosomally GFP-tagged *ENA1* locus |
| ENA1-pFA6-comp-Nter | ATAATCTCGTTTGCCATGC | Verification of specific *ENA1* integration GFP tagging |
| ENA1-pFA6-comp-Cter | AACTTTACAAGTGTGGAAAGAG | Verification of non-specific *ENA* cluster integration GFP tagging |
| pFA6a_GFP_rev | TGAAAAGTTCTTCTCCTTTACT | Verification of non-specific and specific *ENA* cluster integration GFP tagging |
| ENA1_f_RTPCR_2 | CAGCCCAGTCAAGGAATTTT | *ENA1* RT-PCR |
| ENA1_r_RTPCR_2 | TATACGCTTCGAATGGATCG | *ENA1* RT-PCR |
| RT_ACT1_up2 | TGCTGTCTTCCCATCTATCG | *ACT1* RT-PCR |
| RT_ACT1_do2 | ATTGAGCTTCATCACCAAC | *ACT1* RT-PCR |

The underlined sequences are homologous to the plasmids of the pFA6a serie
