# Supplementary material for: Regulation of the Na+/K+-ATPase Ena1 Expression by Calcineurin/Crz1 under High pH Stress: A Quantitative Study
Source: PLoS One. 2016 Jun 30;11(6):e0158424. doi: 10.1371/journal.pone.0158424 (PMC4928930; doi:10.1371/journal.pone.0158424)
Supplement: S1 Text — (DOCX) [file pone.0158424.s002.docx]

**Petrezsélyová *et al*.**

**Regulation of the Na^+^/K^+^-ATPase Ena1 expression by calcineurin/Crz1 under high pH stress: a quantitative study**

**Supplementary Methods for estimation of initial values for the modeling process**

*Estimating total number of Crz1 molecules in a cell.*

The number of Crz1 TF molecules in the cell is approximately constant over time, as shown in Figure 3, which represents the mean from 8 independent experiments. Therefore we assume that total Crz1 remains constant in the model and estimate the number of Crz1 molecules in a cell to be 174 by averaging the eight curves from S1 Fig.

S1 Fig

*Estimating number of nuclear Crz1 molecules over time.*

Fig 2 monitors the cellular localization of Crz1 over the first 20 minutes of the stress response via confocal microscopy. As we do not have single cell measurements of Crz1 amounts, we assumed that the percentage of cells showing nuclear localization of Crz1 is a proxy of the percentage of Crz1 molecules localized in the nucleus within a single cell. Thus, this percentage multiplied by 174 Crz1 molecules/cell gives us an estimation of the number of Crz1 localized in the nucleus though the time course. As the last measure for the localization is 20 min, we assumed that at time 60 min the nuclear concentration of Crz1 is the same that at time 0.

*Estimating Ena1 mRNA concentrations*

Our experimental measurements provide concentrations for Ena1 mRNA in wild type and mutant strains from 0 to 40 min after stress induction. Values from qRT-PCR (Fig 6A), more accurate than those obtained from macroarray analysis (Fig 1) were used for modeling. For fitting and parameter estimation purposes we assume that these concentrations remain approximately constant from 40 to 60 min after stress induction.

*Estimating initial values for the parameters*

Parameter fitting algorithms require initial estimations of parameter values in order to be able to estimate the best fit value. The closer the initial estimate is to the real value, the better the result of the fitting algorithm tends to be.

To estimate the initial values for the rate constants for mRNA synthesis we analyzed the β-galactosidase assay experiments, which provide us a proxy of the capacity of *ENA1* promoter in the presence or absence of Crz1. Fig 6B shows that the β-galactosidase activity reaches a plateau at time 60 in the wild type and *crz1* strains.

The curve for the *crz1* strain is assumed to provide an estimate for *ENA1* promoter activity in the absence of Crz1. Given that we don't have quantitative information about the TFs that regulate this expression, we simply fit the curve to a time dependent B-spline and use this function in the ordinary differential equation models.

Crz1-dependent *ENA1* promoter activity can be estimated by subtracting the curve for the *crz1* strain from that for the wild type. When this is done, Crz1 appear to be responsible for modulating a little less than half of the *ENA1* promoter activity. The initial slope of the subtracted curve is used as the initial estimation for the rate constant α1. Initial values for apparent kinetic orders were set to 1.

For the mutant strain, **α1 Crz1n^g1^** =0
